# Effect of Solution Composition Variables on Electrospun Alginate Nanofibers: Response Surface Analysis

**DOI:** 10.3390/polym11040692

**Published:** 2019-04-16

**Authors:** Janja Mirtič, Helena Balažic, Špela Zupančič, Julijana Kristl

**Affiliations:** Faculty of Pharmacy, University of Ljubljana, Aškerčeva 7, 1000 Ljubljana, Slovenia; janja.mirtic@ffa.uni-lj.si (J.M.); helenabalazic906@gmail.com (H.B.); spela.zupancic@ffa.uni-lj.si (Š.Z.)

**Keywords:** nanofibers, electrospinning, alginate, polyethylene oxide, conductivity, viscoelastic properties, response surface methodology

## Abstract

Alginate is a promising biocompatible and biodegradable polymer for production of nanofibers for drug delivery and tissue engineering. However, alginate is difficult to electrospin due to its polyelectrolyte nature. The aim was to improve the ‘electrospinability’ of alginate with addition of exceptionally high molecular weight poly(ethylene oxide) (PEO) as a co-polymer. The compositions of the polymer-blend solutions for electrospinning were varied for PEO molecular weight, total (alginate plus PEO) polymer concentration, and PEO proportion in the dry alginate–PEO polymer mix used. These were tested for rheology (viscosity, complex viscosity, storage and loss moduli) and conductivity, and the electrospun nanofibers were characterized by scanning electron microscopy. One-parameter-at-a-time approach and response surface methodology (RSM) were used to optimize the polymer-blend solution composition to obtain defined nanofibers. Both approaches revealed that the major influence on nanofiber formation and diameter were total polymer concentration and PEO proportion. These polymer-blend solutions of appropriate conductivity and viscosity enabled fine-tuning of nanofiber diameter. PEO molecular weight of 2–4 million Da greatly improved the electrospinnability of alginate, producing nanofibers with >85% alginate. This study shows that RSM can be used to design nanofibers with optimal alginate and co-polymer contents to provide efficient scaffold material for regenerative medicine.

## 1. Introduction

Polymer nanofibers represent a very promising nanostructured material for biomedical applications, such as advanced drug delivery systems [1,2], wound dressings [3], vascular grafts, and as a scaffold material in regenerative medicine [4,5,6]. Trends in regenerative medicine are progressing toward the use of biomaterials as tissue scaffolds that can promote endogenous healing on their own, without the need for delivery of cells or therapeutics. The extracellular matrix (ECM) influences all aspects of cell behavior, and it is usually damaged or lost in disease or injury. Biomaterials that resemble the ECM and its mechanical properties, architecture, and degradation rate would promote cell adhesion and infiltration, and thus provide improved tissue responses than can be obtained with the delivery of only cells to a damaged tissue [7].

Nanofibers represent a perfect candidate material, with their ultrahigh surface area and tunable morphology and porosity, and with bioadhesion and structure similar to the ECM. Nanofibers can enable the construction of a three-dimensional tissue scaffold of suitable thickness, strength, and mesh size for adequate cell infiltration [4,8]. They can be prepared from polymers of synthetic (e.g., poly(ε-caprolactone), poly(vinyl alcohol), polyurethane, copolymer poly(lactide-co-glycolide)) or natural (e.g., collagen, chitosan, gelatin, silk fibroin) origins [9]. However, biomedical applications require biocompatible and biodegradable nanofibers that will not promote adverse immunogenic responses in the host [10]. This can be achieved with nanofibers made from natural polymers, which includes polysaccharides [9].

Natural biopolymers are gaining particular interest also due to the importance of the eco-friendliness and sustainability of the products, as opposed to the use of fossil-fuel-derived plastics [11]. Another advantage of polysaccharides is their water solubility, as this provides an additional contribution to environmental protection, along with lower monetary cost, and increased practical application. One such polymer is alginate (alginic acid and its sodium salt), which is produced from brown algae (e.g., *Laminaria* sp., *Ascophyllum nodosum*, *Macrocystis pyrifera*) [12]. Alginate is a linear co-polymer that consists of two different types of monomers: α-L-guluronic and β–D–mannuronic acid. These are connected through β-1,4-glycosidic bonds, forming a structure that resembles that of a glycosaminoglycan, the main component of the ECM [13,14].

Electrospinning has been recognized as a cost-effective, versatile, and useful laboratory method for the production of fibrous mats with large surface areas, and also with the possibility of scaling up [15,16,17,18,19]. In drug delivery, it has been investigated for the drug or probiotic incorporation into nanofibers [2,18,20,21], for the production of amorphous solid dispersions [22], taste masking [23] and as an alternative to the lyophilisation for the drying of the therapeutic proteins [24]. Electrospinning is also a rare nonthermal method that is suitable for the preparation of nanofibers from biopolymers, as these are sensitive to high temperatures [11]. The basis of electrospinning is the application of a high voltage to a polymer solution that is being pushed through a needle. Once the voltage exceeds the surface tension of a drop at the end of the needle, a Taylor cone is formed. The electrohydrodynamic cone jet then travels toward a grounded collector, and undergoes stretching and thinning, such that nanofibers are formed [25]. The solvent also evaporates during the process, so solid nanofibers are collected [26].

Although polysaccharides are very attractive excipients for nanofiber production, as has been widely investigated, it has generally not been possible to achieve high polysaccharide contents in nanofibers. The main problem is the difficulty of electrospinning alginate, as for charged polysaccharides in general. The challenges of electrospinning of alginate can be ascribed to its polyelectrolyte nature and chain conformation characteristics [27,28,29]. Charged alginate chains will repel each other due to their repulsive electrostatic interactions [30,31,32]. They are also extended and rigid in water due to their diaxial linkages and stabilization by hydrogen bonds [28,33,34] These hydrogen bonds are forming a gel network, which prevents effective chain entanglement and hinders jet elongation during electrospinning. To obtain nanofibers, the polymer jet must remain unbroken until it reaches the collector plate. These appear to be the reasons why electrospinning of pure alginate in aqueous solution is particularly difficult, and indeed generally impossible. It has thus become apparent that rheology measurements of polyelectrolyte solutions represent a necessary tool to predict potential nanofiber formation [35].

To solve these problems of electrospinning of alginate, a number of studies have reported the combined use of another, noncharged polymer [28,29,32,35]. It would appear that addition of such polymers can lower the repelling forces between the polysaccharide chains, and thus enable nanofiber formation. The most commonly used polymers for nanofiber formation with natural polymers are poly(ethylene oxide) (PEO) and poly(vinyl alcohol), as nonionogenic, linear, water-soluble, and flexible polymers that can significantly improve polysaccharide electrospinnability [28,29]. We specifically selected PEO here because it can modulate the repulsive forces among polyanions while improving the flexibility of alginate chains [36]. Increasing the flexibility and polymer-chain entanglements of the otherwise rigid and extended chains of alginate molecules in aqueous solution is essential for the formation of a continuous jet, to form nanofibers with homogeneous morphology and diameter. Polymer solutions with inadequate entanglements might form beaded fibers or droplets, such as those produced with low polymer concentrations or with low polymer viscoelasticity or molecular weight (M_w_) [30,33].

The electrospinning of sodium alginate from aqueous solutions is thus a challenge, and there are still unsolved questions as to how we can increase and improve such nanofiber production. There are also more recent studies that have dealt with the preparation of alginate nanofibers where high M_w_ PEO (e.g., 900 kDa) was selected as the co-polymer to improve the functional properties of alginate fibers [36]. However, to produce alginate nanofibers, there was the need for >50% PEO in the dry polymer blend used in their production. PEO is not of natural origin, and therefore there is always the tendency to add the smallest amounts possible. However, there are many parameters of a polymer-blend solution that can be varied and will have an influence on nanofiber formation. To better define this situation, most studies have focused on systematically changing only one factor at a time, which fails to take into account any interactive effects between different factors.

For such responses that might be influenced by several variables, the use of multivariate statistical techniques has great potential, such as response surface methodology (RSM) [37]. RSM is a collection of mathematical and statistical techniques that are based on the fit of a polynomial equation to experimental data. The equations obtained can describe the effects of multiple controllable input variables and their interactions for one or many observable output responses. To date, only a few studies have used such a multivariate approach to investigate the potential of their experimental design for the development of nanofibers [38].

The main objective of the present study was to produce electrospun nanofiber mats from alginate–PEO blends with as high an alginate content as possible. Our goal was to use these two polymers, alginate and PEO, without any surfactant or co-solvent, as these have the potential for toxic effects. To identify the main compositional effects on nanofiber formation, the experimental design was set up with three variables (i.e., independent variables) that defined the composition of the polymer-blend solution for the electrospinning, and these were systematically varied:

(i) PEO M_w_: 2, 4 and 8 million (M)Da;
(ii) Total polymer (alginate plus PEO; *w* + *w*) concentration: 2.5%, 3.5%, 4.5% (*w*/*w*);
(iii) PEO proportion of the dry polymer mix: 1% to 15% PEO (to 100% with alginate; *w*/*w*).

The PEOs used in the present study had higher M_w_ than PEOs that are more traditionally used, and we systematically investigated their influence on the properties of the polymer-blend solution. We hypothesized that we could use a smaller amount due to the higher M_w_ and that PEO chain length has an important role in electrospinning. As a second variable for the polymer-blend solution, we took the total polymer concentration (alginate plus PEO), and, as a third, the PEO proportion of the total polymer in the dry polymer mix (of alginate plus PEO) that was used to form the polymer-blend solution.

To determine how these three factors affected the electrospinnability of the polysaccharide solutions, and to explain the effects on chain entanglements in the polymer-blend solution, we measured the rheological properties (viscosity, storage and loss moduli) and conductivity of the polymer-blend solutions, and examined the morphology of the resultant nanofibers. All of the data were analyzed using a nonmodel approach as well as using RSM, to identify the most significant composition parameters of the polymer-blend solution and their interactive effects on the electrospinning. This is a new approach to the composition design of alginate–PEO polymer-blend solutions, which was followed to predict the nanofiber formation and characteristics across a larger experimental space, using a faster methodology, to obtain deeper understanding of this system.

## 2. Materials and Methods

### 2.1. Materials

Sodium alginate (M_w_, 1.38 × 10^5^ g/mol [138 kDa]; Protanal LF 10/60) was from FMC BioPolymer (Haugesund, Norway), and was defined by the manufacturer as 65%–75% α-l-guluronate and 25%–35% β-d-mannuronate. The PEOs of three different M_w_ were all obtained from Sigma-Aldrich (Steinheim, Germany): 2 MDa, 4 MDa and 8 MDa. Purified water was used for preparation of the polymer-blend solutions.

### 2.2. Experimental Design

**Figure 1:**
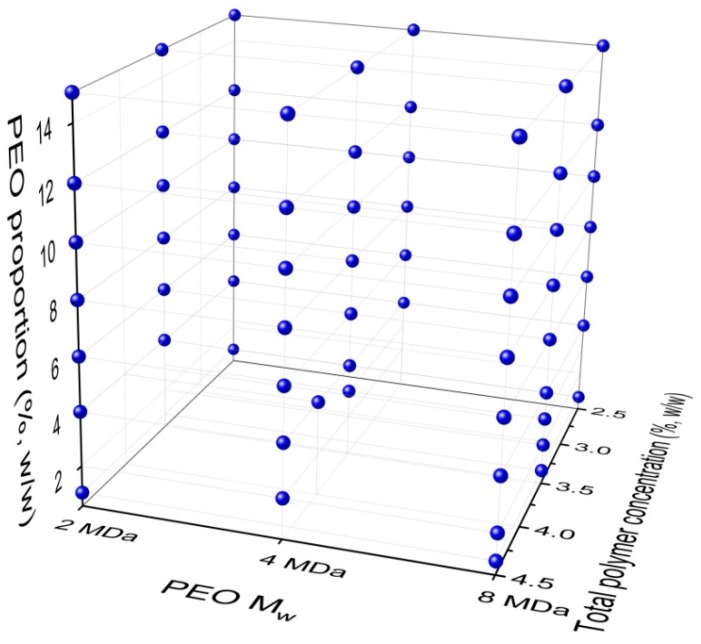
Three-dimensional representation of the experimental design space of the polymer-blend solution compositions for the nanofiber formation. The three components varied were: PEO M_w_; total polymer (alginate plus PEO) concentration; and PEO proportion in the dry alginate–PEO polymer mix used for the polymer-blend solution.

For the experimental design, the 65 different polymer-blend solutions investigated are illustrated in Figure 1. These were prepared by varying the PEO M_w_ (Figure 1, *x*-axis), the total polymer concentration (Figure 1, *y*-axis), and the PEO proportion in the dry alginate–PEO polymer mix (Figure 1, *z*-axis), as the three independent variables. The required masses of the dry polymer mixes were slowly added into the water, which was then left stirring (20 rpm; magnetic stirrer) for 24 h, to obtain homogenous solutions. These solutions were then left standing long enough for any bubbles to leave the solution. Each polymer-blend solution was thoroughly characterized and then used for the electrospinning.

### 2.3. Characterization of the Polymer Solutions

The conductivity (*κ*) of the polymer-blend solutions for electrospinning was measured using a conductivity meter (MA 5964; Iskra, Ljubljana, Slovenia) at room temperature, with an electrode conductivity constant of 0.7265/cm.

The rheology characterization included rotational tests to determine the bulk viscosity (*η*) and oscillatory tests to determine the storage (*G*′) and loss (*G*″) moduli and the complex viscosity (η*). A rheometer (Physica MCR 301; Anton Paar, Graz, Austria) was used with a cone-plate measuring system (CP50-2; cone radius, 24,981 mm; cone angle, 2.001; sample volume, 1.15 mL) at constant temperature of 25.0 ± 0.1 °C. The shear rate during the rotational tests ranged from 2/s to 100/s. The *η* was calculated according to Equation (1):

(1) $$\eta =\tau_{c}/\dot{\gamma }$$

where *τ_c_* is the shear stress and $\dot{\gamma }$ is the shear rate. The oscillatory shear measurements were performed at an amplitude of 1%, which is within the linear viscoelastic region (as determined in prior amplitude sweep experiments), and a frequency from 0.1/s to 100/s. The storage modulus (*G*′) and loss modulus (*G*″) were calculated according to Equations (2) and (3):

(2) $$G^{\prime }=(\tau_{a}/\gamma_{a})\times \cos\delta ,$$

(3) $$G^{\prime \prime }=(\tau_{a}/\gamma_{a})\times \sin\delta ,$$

where *τ_a_* is the shear stress, *γ_a_* is the deformation, and *δ* is the phase shift angle. The complex viscosity (η^*^) was calculated according to Equation (4):

(4) $$\left|\eta^{*}\right|=G^{*}/\omega ,$$

where G* is the complex shear modulus, which was calculated according to Equation (5):

(5) $${\left(G^{*}\right)}^{2}={\left(G^{\prime }\right)}^{2}+{\left(G^{\prime \prime }\right)}^{2}$$

The RSM analysis was performed on parameters (*η*, *G*′, *G*″, η*) at the determined collection points. The *η* values were collected at a shear rate of 2/s, where the load of the system minimally influences the *η*. The values of both the storage and loss moduli were collected at low frequency (0.158/s), where they have the most dramatic differences, and the values of η^*^ were collected at high frequency (100/s).

### 2.4. Electrospinning

The homogeneous polymer-blend solutions were used to fill a 5-mL disposable syringe that was fixed into the electrospinning machine (Fluidnatek LE100; BioInicia SL, Valencia, Spain). An electric field of 23 ± 6 kV was applied between the needle tip and a grounded flat collector that was covered with aluminum foil. The distance between the syringe tip and the grounded flat collector was 20 cm, and the flow rate of the electrospinning solution was 700 ± 200 μL/h. All of the electrospinning was conducted in a climatic chamber with a controlled environment of 37 ± 0.5 °C and 19% ± 2% relative humidity.

### 2.5. Characterization of Nanofibers Using Scanning Electron Microscopy

The samples collected were fixed with double sided adhesive and conductive tape onto the stubs for the scanning electron microscopy (SEM), and the images were collected using high resolution SEM (235 Supra 35VP-24-13; Carl Zeiss, Jena, Germany) operated at the increasing voltage of 1 kV at different magnifications. No conductive coating was applied before the imaging. The diameters (*d*) of the nanofibers were measured and averaged over at least 50 nanofibers in the SEM images within representative microscopic fields, using the Image J software (1.51j8, National Institutes of Health, Bethesda, MD, USA).

### 2.6. Statistical Analysis

The OriginPro17 software (Originlab Corporation, Northampton, MA, USA) was used for creation of the graphs for the nonmodel data analysis. The Minitab 17 statistical software (Minitab Inc., State College, PA, USA) was used for evaluation of the relationships between the independent variables (C1, PEO M_w_; C2, total polymer concentration [%; *w*/*w*]; C3, PEO proportion in the dry polymer mix [%; *w*/*w*]) and the output responses (*κ*, *η*, η*, *G*′, *G*″, *d*) using RSM. Analysis was performed using coded coefficients and design with equations and graphs are presented in uncoded units. For surface response analysis, quadratic models were fitted. An exemplary equation is given in Equation (6):

(6) $$\begin{array}{l} Y=a_{0}+a_{1}*X_{1}+a_{2}*X_{2}+a_{3}*X_{3}+a_{11}*X_{1}^{2}+a_{22}*X_{2}^{2}\\ +a_{33}*X_{3}^{2}+a_{12}*X_{1}*X_{2}+a_{13}*X_{1}*X_{3}+a_{23}*X_{2}*X_{3} \end{array}$$

where *Y* represents the response, *X* represents the independent or dependent variables, *a*_0_ is a constant, and *a*_i_, *a*_ii_ and *a*_ij_ are the linear, quadratic, and interactive coefficients, respectively. The goodness of fit of each model was evaluated by coefficient determination: R^2^ and R^2^ (adjusted). R^2^ indicates the percentage of the total variations in the experimentation, and R^2^ (adjusted) indicates the significance of the model, where values closer to 1 indicate better model prediction. Contour plots were used for visualization of the equations. A response optimizer was used to describe the effects of each variable on the selected responses, and was visualized as response optimization plots.

## 3. Results

First, we took the traditional approach of a one-factor-at-a-time data analysis of the experimental data obtained without using any models. The data are presented as SEM images in combination with the rheology data of the electrospun solutions, to visualize and predict correlations between solution composition, solution characteristics, and nanofiber morphology and diameter. The data obtained are relatively complex, as there were three independent variables in the composition, and they all interactively affected the solution parameters. Thus, a more advanced analysis of the data was needed. As such, RSM analysis was applied and the results are presented in Section 3.2.

### 3.1. Nonmodel Approach to Analyze Alginate Nanofiber Formation

Formulations for which we were able to obtain a sample on the collector (although not necessary as nanofibrous mats) were selected, with the SEM images for visual presentation and connected with the rheology data (*η*, η*; *G*′, *G*″). For all of the cases presented, two of the composition variables were fixed and only one was varied. The idea was to understand the separate effects of PEO M_w_, total polymer concentration, and PEO proportion in the dry polymer mix on the electrospinning of the alginate–PEO polymer-blend solution and the morphology of the nanofibers.

#### 3.1.1. Effects of PEO Molecular Weight

**Figure 2:**
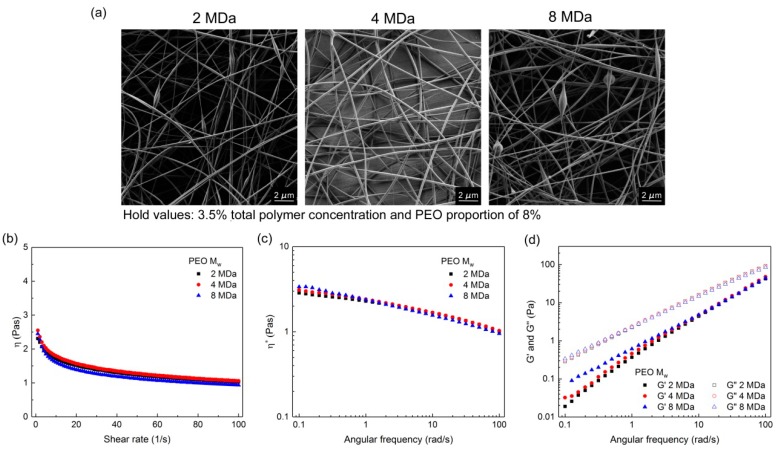
(**a**) scanning electron microscopy images of nanofibers obtained from the polymer-blend solutions and three different PEO M_w_ (as indicated), with 3.5% total polymer concentration and PEO proportion of 8%; (**b**–**d**) corresponding viscosities (*η*) (**b**); complex viscosities (η*) (**c**); and storage (*G*′) and loss (*G*″) moduli (**d**).

Figure 2 shows the SEM images of one set of polymer-blend solutions where nanofibers were obtained for all three of the PEO M_w_ using the 3.5% total polymer concentration (in the polymer-blend solution) and the PEO proportion of 8% (in the dry polymer mix used in the polymer-blend solution; i.e., with alginate of 92%). Although the PEO M_w_ was increased, it did not show any correlation with nanofiber diameter. The nanofiber diameters obtained were 148 ± 74 nm, 234 ± 67 nm and 178 ± 46 nm for the nanofibers prepared from the 2, 4, and 8 MDa PEO, respectively. In addition, all of the rheological data were similar between these different compositions. In comparison, with the 2.5% and 4.5% total polymer concentrations with the PEO proportion of 15% (Supplementary Information Figures S2 and S3), the bulk rheology looked similar across all of these formulations, with a tendency for a small increase with increasing PEO M_w_. However, in these cases when the higher PEO proportion in the dry polymer mix was used, the electrospinning of solutions with 8 MDa PEO resulted in microfiber formation (e.g., Supplementary Information Figures S2, S3 and S5).

The data presented here are only from the cases where all three of PEO M_w_ formulations could be analyzed by SEM, as many others could not be, with further details in Supporting Information Figure S1. In many cases, the formulations with 8 MDa PEO could not be electrospun at all, and no samples were obtained. Thus, even from these initial observations, we recognized that 8 MDa PEO was a less suitable co-polymer for the formation of alginate–PEO nanofibers. 

#### 3.1.2. Effects of Total Polymer Concentration

**Figure 3:**
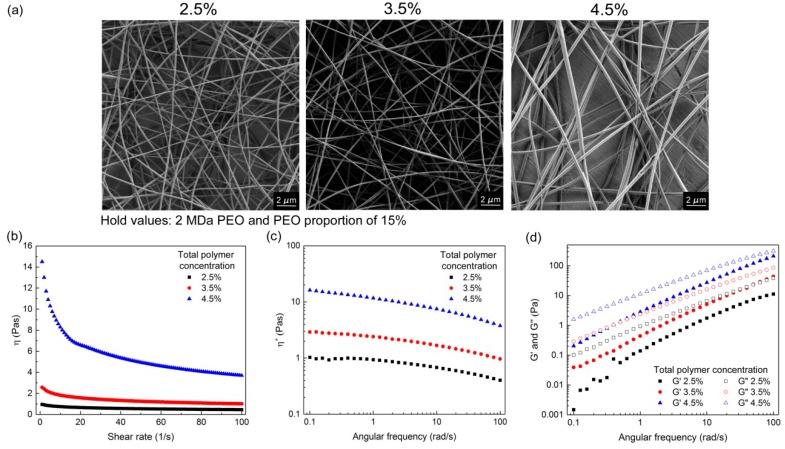
(**a**) scanning electron microscopy images of nanofibers obtained from polymer-blend solutions with increasing total polymer concentrations (as indicated), with 2 MDa PEO and PEO proportion of 15%; (**b**–**d**) corresponding viscosities (*η*) (**b**); complex viscosities (η*) (**c**); and storage (*G*′) and loss (*G*″) moduli (**d**).

**Figure 4:**
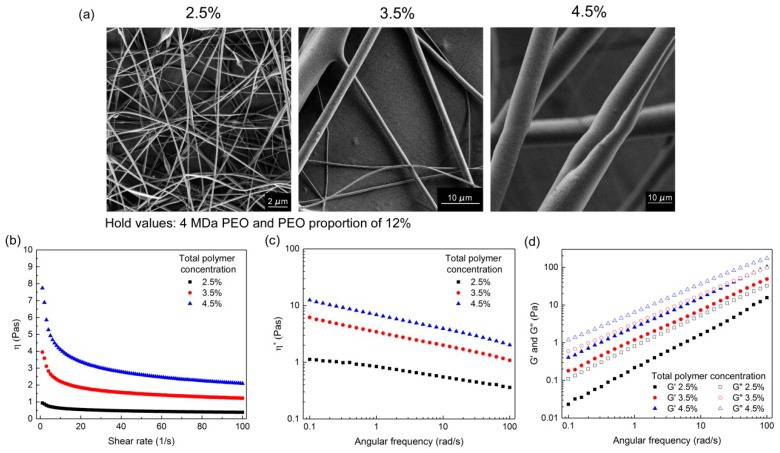
(**a**) scanning electron microscopy images of nanofibers obtained from polymer-blend solutions with increasing total polymer concentrations (as indicated), with 4 MDa PEO and PEO proportion of 12%; (**b**–**d**) corresponding viscosities (*η*) (**b**), complex viscosities (η*) (**c**), and storage (*G*′) and loss (*G*″) moduli (**d**).

The effects of increasing the total polymer concentrations in the polymer-blend solutions (2.5%, 3.5%, 4.5%) are presented for all three PEO M_w_, as the following: 2 MDa PEO with PEO proportion of 15% (Figure 3), 4 MDa PEO with PEO proportion of 12% (Figure 4), and 8 MDa PEO with PEO proportion of 4% (Supplementary Information Figure S4). Increasing the total polymer concentration resulted in increases in all of the rheological parameters, and there were different effects on the nanofiber morphology. These morphology effects can be summarized as increased nanofiber diameter (Figure 3), formation of microfibers (Figure 4), or shift from beaded nanofibers to smooth and beadless nanofibers (Supplementary Information Figure S4). In more detail, the polymer-blend solutions with total polymer concentrations 2.5%, 3.5%, and 4.5% shown in Figure 3 resulted in nanofibers with increasing diameters: 134 ± 32 nm, 149 ± 31 nm, and 288 ± 46 nm, respectively. For the data shown in Figure 4, nanofibers were obtained only with the 2.5% polymer concentration (diameter, 186 ± 45 nm), while, at the higher total polymer concentrations, microfibers were formed (diameter, up to 10 μm). However, the rheological data are not sufficient to predict the formation of nanofibers or microfibers. Indeed, in some cases, the viscosity and complex viscosity values of the polymer-blend solutions were similar, while the products that resulted from the electrospinning of these solutions were very different. For example, making a comparison of the formulations with 4.5% total polymer concentration from Figure 3 and Figure 4, it can be seen that their values of *η*, η*, *G*′, and *G*″ are similar; however, the former resulted in nanofiber formation, and the latter in microfiber formation. This shows that the nanofiber characteristics cannot be attributed to this single composition parameter, and thus that the interaction effects of the formulation compositions must also be taken into account.

#### 3.1.3. Effects of PEO Proportion in the Dry Polymer Mix

**Figure 5:**
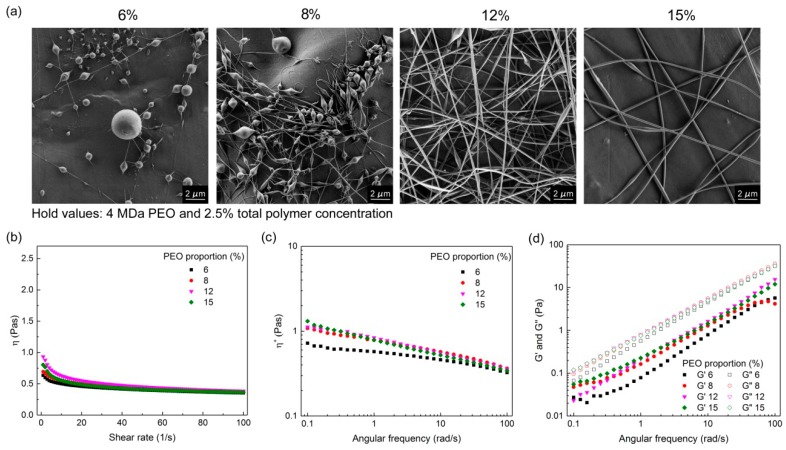
(**a**) scanning electron microscopy images of nanofibers obtained from polymer-blend solutions formed with different PEO proportions in the dry polymer mix (as indicated), with 4 MDa PEO and 2.5% total polymer concentration; (**b**–**d**) corresponding viscosities (*η*) (**b**); complex viscosities (η*) (**c**), and storage (*G*′) and loss (*G*″) moduli (**d**).

Increases in the PEO proportion in the dry polymer mix were made to potentially improve the electrospinnability of the resulting polymer-blend solutions. This was indeed the case, as can be seen for the formulations shown in Figure 5, which were based on 4 MDa PEO M_w_ and 2.5% total polymer concentration in the polymer-blend solutions. For the lower PEO proportions of 6% and 8%, there was formation of beaded nanofibers, whereas smooth nanofibers (diameter, ~190 nm) were produced with the higher PEO proportions of 12% and 15%. This trend was also confirmed for nanofibers prepared from the 2 MDa and 8 MDa PEO (Supplementary Information Figures S5 and S6). However, the solution parameters (i.e., total polymer concentration, PEO proportion) at which the beadless nanofibers were formed were specific for each PEO M_w_. Conversely, there were no effects seen for increases in the PEO proportion on nanofiber diameter within the individual cases.

Here, although the values of *η*, η*, and *G*″ are similar between the formulations within this group, the storage modulus (*G*′) was the most powerful predictor of the formation of beaded nanofibers. When the curves of the storage modulus started to decrease with increasing angular frequency (Figure 5d), beaded nanofibers were formed (Figure 5a). Thus, elasticity is necessary for nanofiber formation, as its lack results in unsuitable elongation of the polymer chains and instability of the spinning jet.

We can conclude from this nonmodel data analysis that the composition of these alginate–PEO polymer-blend solutions had major effects on the electrospinning and morphology of the nanofibers obtained. The main observations from this analysis are: (a) PEO M_w_ does not show any straightforward correlations with nanofiber diameter; (b) increasing the total polymer concentration results in increases in the nanofiber diameters; (c) increasing the PEO proportion in the dry polymer mix for the polymer-blend solutions results in less beaded and smoother nanofibers; and (d) the behavior of the storage modulus appears to be the most indicative parameter for nanofiber formation. However, for all of these solutions, the characteristics are interconnected, and this one-factor-at-a-time approach is not sufficient for the simultaneous analysis of these multiple parameters.

### 3.2. Response Surface Methodology to Analyze the Alginate Nanofiber Formation

The major advantage of RSM over the one-factor-at-a-time approach is that it allows evaluation of the effects of many independent variables and their interactions on one or more observable responses. The measured polymer-blend solution characteristics and the nanofiber diameters are treated as the responses of interest in this analysis, and they are influenced by all three of the polymer-blend solution composition parameters (independent variables). Surface response model equations that are based on the fitting of the polynomial equations to the experimental data were used and are presented here.

The first set of modeling describes the influence of the polymer-blend solution compositions on the characteristics of these solutions. PEO M_w_ (C1), total polymer concentration (C2), and PEO proportion in the dry polymer mix (C3) are treated as the independent variables, and conductivity (*κ*), bulk viscosity (*η*) and complex viscosity (η^*^) as the dependent variables. In the next step, only the formulations of the polymer-blend solutions that resulted in nanofiber formation (28 of the 65) were analyzed, with both the independent and dependent (*κ*, *η*, *G*′, *G*″) variables correlated to the nanofiber diameters (*d*).

Contour plots are used to visualize the RSM, to show how a response variable relates to two continuous independent variables based on the model equations. The held values for the data of two out of the three variables in the contour plots are always the same: 4 MDa PEO M_w_, 3.5% total polymer concentration, and PEO proportion of 8%. The optimization plots were constructed to show how the corresponding response variable changes as a function of one of the variables, while all of the others remain fixed.

#### 3.2.1. Polymer Blend Solution Properties Dependence on Composition

##### (a) Conductivity

The response values for the conductivities were fitted with a quadratic model, which resulted in Equation (7) (R^2^ = 0.6603, R^2^(adjusted) = 0.6047):

(7) $$\begin{array}{l} \kappa =0.31-0.000001*C1+3.51*C2-0.331*C3-0.417*{\left(C2\right)}^{2}+0.0065*{\left(C3\right)}^{2}\\ +0.0455*C2*C3 \end{array}$$

**Figure 6:**
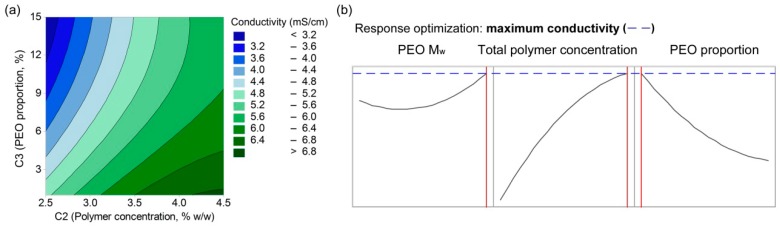
(**a**) contour plot of the polymer-blend solution conductivity as a function of PEO proportion and total polymer concentration; (**b**) response optimization plots of the polymer-blend solution conductivity as a function of PEO M_w_, total polymer concentration, and PEO proportion.

In terms of the conductivity, three linear terms (C1, C2, C3), two quadratic terms (C2, C3) and one interaction term (between C2 and C3) were identified as important. The values in Equation (7) indicate that the conductivity of the polymer-blend solution increases with increasing total polymer concentration (C2) as well as with higher levels of alginate in the polymer-blend solution (i.e., lower PEO proportions; C3). Both of these are related to the amount of alginate chains present, as they mainly contribute to the charge, as well as to the presence of counter ions (Na^+^) that are released once the alginate is dissolved in the water. Parameters C2 and C3 show positive interactive effects on conductivity. Contrarily, PEO M_w_ (C1) has negligible effects. The set model (Equation (7)) is also presented as a contour plot (Figure 6a) and as response optimization plots (Figure 6b), which show how the conductivity changes as a function of one of the independent factors. These plots confirm the greatest effects of the polymer concentration (C2), lower effects of PEO proportion (C3), and the minor effect of PEO M_w_ (C1) on these polymer-blend solution conductivities.

##### (b) Viscoelastic Parameters

The modeling of the bulk viscosities measured at a shear rate of 2/s as a response value with a quadratic model resulted in Equation (8) (R^2^ = 0.9180, R^2^(adjusted) = 0.9046):

(8) $$\eta =1.454-0.876*C2-0.1464*C3-0.2261*{\left(C2\right)}^{2}+0.0047*{\left(C3\right)}^{2}+0.0349*C2*C3$$

**Figure 7:**
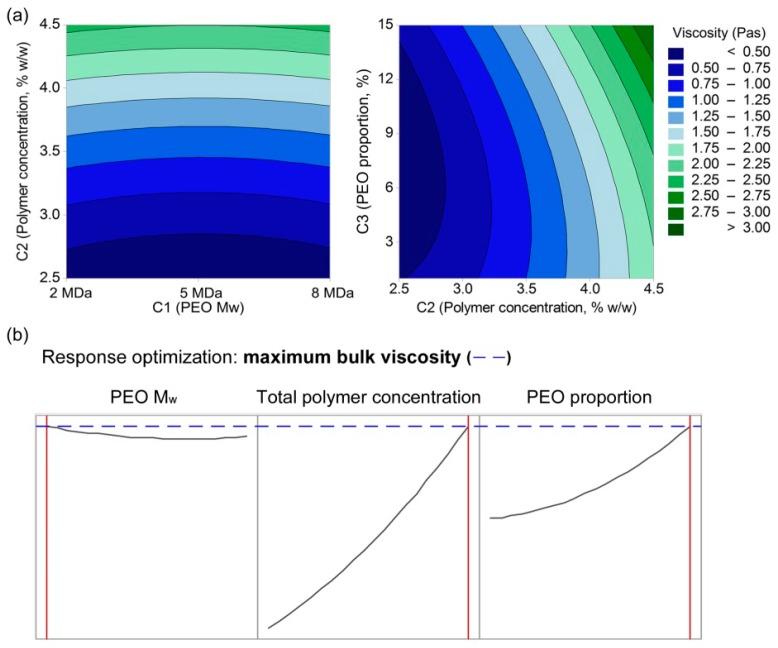
(**a**) contour plots of the polymer-blend solution bulk viscosity as a function of total polymer concentration and PEO M_w_ (left) and total polymer concentration and PEO proportion (right); (**b**) response optimization plots of the polymer-blend solution bulk viscosity as a function of PEO M_w_, total polymer concentration, and PEO proportion.

Linear, quadratic, and interaction terms were obtained here only for the C2 and C3 parameters, which shows their interactive effects on the viscosity of the polymer-blend solutions. The contour plots (Figure 7a) and response optimization plots (Figure 7b) revealed that the bulk viscosity of the polymer-blend solutions increases with increases in the total polymer concentration, whereas the PEO proportion has a minor role, and PEO M_w_ does not have any effect on the viscosity. The modeling of the complex viscosity and loss modulus (Supplementary Information Figures S7 and S8) revealed that the correlations are in line with the modeling of the bulk viscosity, whereas the modeling of the storage modulus does not result in any relevant model (Supplementary Information Equation (S2)).

#### 3.2.2. Influence of Solution Composition and Solution Characteristics on Nanofiber Diameter

##### (a) Nanofiber Diameter Dependence on Solution Composition

The correlation between the composition parameters and the nanofiber diameter resulted in Equation (9) (R^2^ = 0.6673, R^2^(adjusted) = 0.5009):

(9) $$\begin{array}{l} d=2936-0.0007*C1-2623*C2-785*C3-312*{\left(C2\right)}^{2}+17.8*{\left(C3\right)}^{2}\\ +0.00018*C1*C2+0.0001*C1*C3+118*C2*C3 \end{array}$$

**Figure 8:**
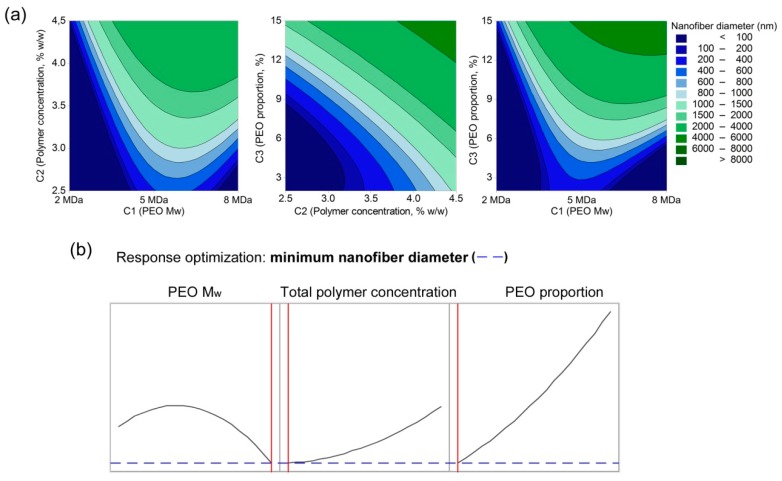
(**a**) contour plots of the resulting nanofiber diameter as a function of total polymer concentration and PEO M_w_ (left), PEO proportion and total polymer concentration (middle), and PEO proportion and PEO M_w_ (right); (**b**) response optimization for the resulting nanofiber diameter as a function of PEO M_w_, total polymer concentration, and PEO proportion.

Three linear terms (C1, C2, C3), two quadratic terms (C2, C3), and all three interaction terms were identified as important influences on the nanofiber diameter. Increasing the total polymer concentration (C2) and PEO proportion in the dry polymer mix (C3) for the polymer-blend solution compositions were reflected in a linear increase in the nanofiber diameter (Figure 8). The model reveals that the thickest fibers are obtained with 4 MDa PEO M_w_ (C1), which might sound contradictory to the expected effects of polymer M_w_. This is also contrary to the nonmodel analysis, where the use of 8 MDa PEO resulted in the formation of microfibers. The model here that contains all of the formulations with 8 MDa PEO results in nanofibers, microfibers, and the less successful formulations with beads in combination with thinner nanofibers (i.e., beads-on-a-string structured nanofibers), where this last greatly contributed to the overall decrease in nanofiber diameter. Therefore, it is advisable to check the model results with the correlations obtained from the nonmodel approach, and also with the experimental observations. We observed that the electrospinning of formulations with 8 MDa PEO was more challenging compared to the formulations with 2 MDa and 4 MDa PEO.

##### (b) Nanofiber Diameter Dependence on Solution Characteristics

• Conductivity and Bulk Viscosity

The nanofiber diameter can be correlated to the solution characteristics using Equation (10) (R^2^ = 0.7477, R^2^(adjusted) = 0.6904) with a better fit compared to the polymer-blend solution composition parameters alone (Equation (9)):

(10) $$d=-963-816*\kappa +3537*\eta +182.7*\kappa^{2}+124.7*\eta^{2}-665*\kappa *\eta$$

**Figure 9:**
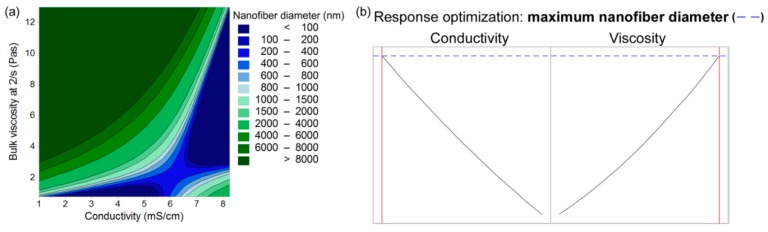
(**a**) contour plot of the resulting nanofiber diameter as a function of the polymer-blend solution conductivity and bulk viscosity; (**b**) response optimization of the resulting nanofiber diameter as a function of the polymer-blend solution conductivity and bulk viscosity.

The linear, quadratic and interaction terms describe the interactive effects of the polymer-blend solution conductivity and viscosity on the nanofiber diameter. Separately, higher conductivity and lower viscosity results in thinner nanofibers, as seen from the response optimization plots (Figure 9b). The relationship between the conductivity and viscosity is interactive (Equation (10)) and complex, as can be seen from the contour plot (Figure 9a). Nanofibers can only be obtained in a very specific narrow region of conductivities and viscosities (Figure 9a, blue area). The dark blue area in Figure 9a (*d* < 100 nm) does not necessarily represent the thinnest nanofibers, but rather those obtained in an unstable process where the beads-on-a-string structured nanofibers were formed.

• Storage and Loss Moduli

The nonmodel approach indicated the importance of the viscoelastic properties of the polymer-blend solution for nanofiber formation. Therefore, we correlated the nanofiber diameter (*d*) with the storage and loss moduli using Equation (11) (R^2^ = 0.5252, R^2^ (adjusted) = 0.4173):

(11) $$d=-496+358*G^{\prime }+22*G^{\prime \prime }-19{\left(G^{\prime }\right)}^{2}-8.2*{\left(G^{\prime \prime }\right)}^{2}+23.1*G^{\prime }*G^{\prime \prime }$$

**Figure 10:**
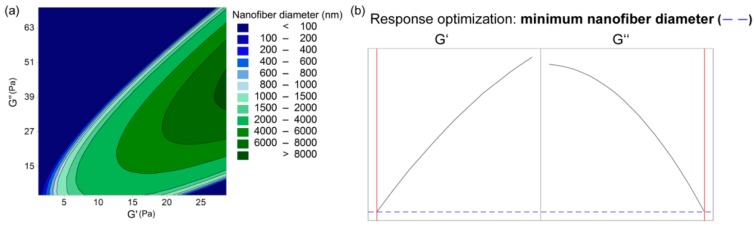
(**a**) contour plot of the resulting nanofiber diameter as a function of the polymer-blend solution storage (G′) and loss (G″) moduli; (**b**) response optimization plots of the nanofiber diameter as a function of the polymer-blend solution storage and loss moduli.

The storage and loss moduli and their interactions have effects on the nanofiber diameter. The parameters are reversely related to the nanofiber diameter, as thinner nanofibers are obtained with lower storage modulus and higher loss modulus of the polymer-blend solutions (Figure 10). The higher the differences in the values of these moduli, the thinner the nanofibers obtained are; however, this is limited by the storage modulus. The storage modulus must be sufficiently high to obtain nanofibers, otherwise beads or beaded nanofibers are formed (Figure 10a, dark blue area).

From the models obtained, we can draw the following conclusions: (a) There is only a small effect of PEO M_w_ on the polymer-blend solution characteristics and the resulting nanofiber diameter; (b) Increasing the total polymer concentration and the PEO proportion in the dry polymer mix have the biggest effects on the polymer-blend solution characteristics (conductivity and viscosity); (c) Nanofiber diameter depends mainly on the PEO proportion in the dry polymer mix, although the total polymer concentration also has an important role; (d) Both the conductivity and viscosity have interactive effects on the resulting nanofiber diameter; and (e) Viscoelastic properties (G′ and G″) interactively affect the thickness of the resulting nanofibers.

#### 3.2.3. Guidelines for Successful Nanofiber Formation

The models obtained here can also serve us as a guideline for the composition of polymer-blend solutions and as an indication of which polymer-blend solution parameters will result in the appropriate nanofiber formation. In our case, nanofibers from these alginate–PEO blend solutions can be most efficiently obtained with 4 MDa PEO, a total polymer concentration between 3% and 4%, and a PEO proportion in the dry polymer mix of 8% to 12%. The polymer-blend solutions produced would then have the following solution characteristics: conductivity, 4–7 mS/cm; bulk viscosity, 1–6 Pas; G′, 4–10 Pa; and G″, 15–30 Pa.

**Figure 11:**
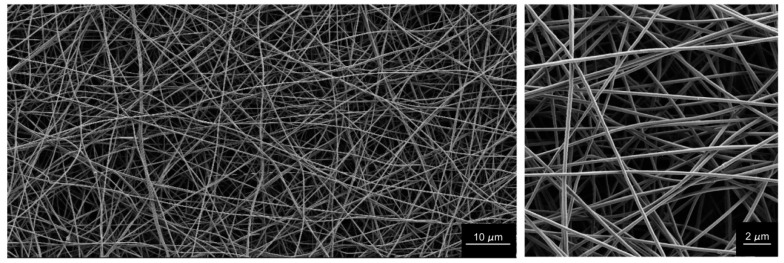
Representative scanning electron microscopy images of nanofibers obtained from the electrospinning of the exemplary polymer-blend solution, with a composition of 4 MDa PEO, 3.5% total polymer concentration, and PEO proportion in the dry polymer mix of 10%. Magnification, 2500× (left); 15,000× (right).

The exemplary formulation had the following composition: 4 MDa PEO, 3.5% total polymer concentration, and PEO proportion of 10%, which means that following the formation of the nanofibers and evaporation of the solvent, there was 90% (*w*/*w*) alginate in the final dry nanofiber mat. The properties of the polymer-blend solution produced were: conductivity, 5.23 mS/cm; bulk viscosity, 2.43 Pas; G′, 4.94 Pa; and G″, 15.6 Pa. Electrospinning of this polymer blend resulted in a very stable electrospinning process and an appropriate nanofiber morphology, with a nanofiber diameter of ~260 nm (Figure 11).

## 4. Discussion

The role of the conductivity and viscoelastic parameters in electrospinning of alginate–PEO blends is indicated by the parallel data analyses for these nonmodel and model-based approaches. The critical factors to improve the electrospinning of these polymer-blend solutions and for fine-tuning of the nanofiber diameter obtained in this study are now discussed and compared with the literature.

### 4.1. Role of Conductivity in Electrospinning of Alginate–PEO Blends

The conductivity of these alginate-PEO blend solutions increased with increased total polymer concentration, and decreased with PEO proportion, regardless of its M_w_ (Figure 6). As such, alginate is the greatest contributor to the solution conductivity due to its anionic nature in water, which is in agreement with previous studies [30]. Alginate chains with ionogenic groups create strong repulsive forces between the polyelectrolyte backbones, and this hinders the formation of a continuous jet during nanofiber formation. Therefore, too high a conductivity of the polymer-blend solution does not result in the formation of nanofibers, but rather in beads or heavily beaded fibers.

The formation of nanofibers can be improved by the addition of PEO, as PEO can shield the negative charges of alginate, and thus lower the conductivity of such polymer-blend solutions. Typically, lower M_w_ PEOs can shield the charges to a greater extent, as short chains have greater mobility in solution and can, therefore, wrap around the alginate negative chains more effectively [36]. In our case, 2 MDa and 4 MDa PEOs had similar effects on the conductivity of the polymer-blend solution, and thus they presumably have similar mobilities. On the other hand, Figure 6b shows that 8 MDa PEO in the polymer-blend solution results in increased conductivity of the solution, which could be related to the decreased PEO mobility due to the longer polymer chains.

However, a decrease in the conductivity of the polymer-blend solution cannot assure improved electrospinnability of alginate. The importance of the PEO M_w_ has been shown previously, although a study was not conclusive in terms of whether these effects on conductivity were amongst the most important ones [29]. As seen from the present study, there is an optimal region of conductance of the polymer-blend solution that must be achieved. However, this parameter is not sufficient to predict the formation of nanofibers, which also depends on the other solution characteristics.

### 4.2. Role of Viscoelastic Parameters in Electrospinning of Alginate–PEO Blends

Pure alginate solutions can form very viscous and conductive fluids, but they cannot be electrospun [29]. Comparing only the zero-shear viscosity values might be misleading, as in the electrospinning process, the polymer solution is exposed to shear stress. Therefore, it is necessary to consider the curves of the viscosity versus the shear rate. Shear thinning behavior is characterized by decreasing viscosity values with increasing shear rate, which was observed for all of the alginate–PEO blend solutions (Figure 2b, Figure 3b, Figure 4b and Figure 5b). This is a typical pseudoplastic behavior of non-Newtonian fluids. It is also characteristic for polymers, where deformation appears in the shear direction (disentanglements), and it can tell us more about the structure of the polymer chains in the solution. Each macromolecule is in its three-dimensional form in solution (higher values of viscosity). When the shear is applied, the macromolecules become oriented parallel to the direction of the shear, which results in elongation of the polymer chains, and, consequently, in a lowering of the flow resistance (which results in decreased bulk viscosity) [35]. The evaluation of the curves of the bulk viscosity versus the shear rate obtained for the different polymer-blend solution compositions reveal that a certain value of the viscosity is needed under high shear stress (>0.5 Pas). However, although this is important, it is not a sufficient indicator for the prediction of nanofiber formation. This study also indicates the strong correlation between the higher bulk viscosity of the polymer-blend solution and the formation of microfibers. However, different polymer-blend solutions with very similar bulk viscosity profiles can result in nanofibers or not. Therefore, the data for the storage and loss moduli were studied to determine their role.

The dependence between these two moduli describes the state of the polymer-blend solution, where G′ > G″ shows that the polymer-blend solution is more gel-like, and for G″ > G′, it is in more liquid state. This latter (G″ > G′) is the case for all of the electrospinnable formulations, which reveals that the polymer-blend solution must behave more like a liquid than a gel [35]. Both the storage and loss moduli increased significantly with the increase in the total polymer concentration (Figure 3 and Figure 4), whereas the PEO proportion in the dry polymer mix (Figure 5) and the PEO M_w_ (Figure 2) resulted in only small increments, which correspond to previously reported data [35]. All of the alginate-PEO blends are characterized with greater plasticity with respect to elasticity across the whole range of frequencies considered—as demonstrated by the higher profiles of the loss modulus with respect to the storage modulus. The model revealed (Figure 10) that higher plasticity (G″) and lower elasticity (G′) result in thinner nanofibers. Plasticity is important as it contributes to the structure transformation, and hence to nanofiber formation. Moreover, the elasticity of the polymer solution is critical in the stage of jet initiation and for appropriate elongation, due to its role in preventing the jet from breaking up. Consequently, polymer-blend solutions with greater elasticity result in a beadless nanofiber structure [39]. We showed that when the storage modulus is decreasing with an increasing angular frequency, beads are formed rather than nanofibers (Figure 5). Therefore, to obtain beadless and smooth nanofibers, it is necessary that there is elasticity also with increasing shear.

### 4.3. Improving the Electrospinning of Alginate-PEO Blends

The main two properties of any polymer solution for successful electrospinning are sufficient chain entanglements and viscoelasticity to stabilize the electrospinning jet [29,30,40]. Using these findings, different approaches have been developed whereby the content of alginate in nanofibers has been increased. One study reported the use of glycerol as a co-solvent, which increases the solution viscosity, improves the flexibility, and enhances the entanglements of the alginate chains. Glycerol disrupts the strong intermolecular and intramolecular hydrogen bonding among the alginate chains, and forms new hydrogen bonds with the alginate chains [33]. PEO and alginate chains can also interact through hydrogen bonding, which contributes to their compatibility in solution, although this interaction alone is not sufficient for a polymer solution to be electrospun. There must be some additional physical interactions between the PEO and the alginate in a blend that make the chain entanglements tighter than for a PEO solution alone [29]. This is supported in that viscosities of blend solutions are not the sum of only the values of the different composite polymers, as the interactions formed between the two polymers additionally increase the viscosity values. For comparison, the values of viscosities of PEO solutions alone (1.05%, 1.5%, Supplementary Information Figure S9) are much lower, almost close to 0, and do not increase the viscosity values of the polymer-blend solutions as much as we observe here. The same conclusions can be drawn from the results of modeling the viscosity with the polymer-blend solution composition parameters (Equation (8); Figure 7), where after the total polymer concentration and the PEO proportion, their interactive effects also contribute to the viscosity.

Bonino et al. [29] showed that PEO chain length has an important role in electrospinning of alginate. Their small amounts (20% in the polymer blend) of 100 kDa PEO already showed some improvements to the electrospinning, due to the increased degree of entanglement. However, large amounts (60%) of 2 kDa PEO had no such effects, as sufficient chain entanglements were not achieved [29]. Similarly, they obtained better electrospinning of their polymer-blend solutions with higher alginate contents when using 400 kDa PEO [35] and 500 kDa PEO [28]. For the present study, our case is unique, as we chose three very high PEOs M_w_: 2 MDa, 4 MDa, and 8 MDa. At any given total polymer concentration, the increasing PEO M_w_ results in increased chain entanglement density, and, consequently, in increased viscosity [41]. At a certain polymer M_w_, a minimum solution concentration is required to obtain beadles nanofibers, which is known as the critical entanglement concentration [29]. This critical entanglement concentration decreases with increasing polymer M_w_, as a result of the longer polymer chain length [30]. However, for alginate alone, the critical entanglement concentration determined is 0.4% (*w*/*v*), which is a lot lower than the concentrations of alginate that are usually tested for electrospinning.

Thus, the traditionally defined entanglement concentration [42] cannot be directly applied to the electrospinnability of alginate [29]. Alginate has a rigid chain structure and extended worm-like chain conformation in solution. These chains cannot entangle with each other to form an interpenetration network, as is the case for the more flexible PEO chains. Alginate chains show closely spaced overlap even in a concentrated regime, and the chains just slide against each other during the electrospinning process. For the system to be efficiently electrospun, the polymer chains must be tight enough with the necessary chain entanglements.

Therefore, high M_w_ PEOs can offer the chain entanglements needed, to form a “cage” that can enclose alginate molecular chains into its large physical network, which will make the packing even tighter than it would be in a PEO alone solution. In the cases of all of the PEO M_w_ used in the present study, sufficient entanglements were reached to lead to successful formation of nanofibers. In this respect, the higher the M_w_ used, the lower the polymer concentration needed for enough entanglements to be formed [41]. However, 8 MDa PEO results in large increases in the rheological parameters, and also has negative effects on the conductivity, due to the lower chain mobility. An example of this can be seen in Figure 2, where the nanofibers with best morphology are formed with 4 MDa PEO (i.e., with sufficient entanglements), but 2 MDa and 8 MDa PEO resulted in less appropriate nanofiber morphologies, due to either not enough entanglements (2 MDa PEO) or not sufficient polymer chain mobility (8 MDa PEO), with both also considered in terms of the other solution composition parameters.

Hence, we propose the use of 2 MDa and 4 MDa PEOs as more relevant for the production of high-polysaccharide-content nanofibers. These are even more useful than the traditionally used 900 KDa PEO, as they enable nanofiber formation with >85% alginate (Figure 11). We also hypothesize that such nanofibers would have improved mechanical properties compared to the ones using lower M_w_ PEOs.

### 4.4. Importance of Nanofiber Diameter and Its Fine Tuning

When considering nanofibers for tissue engineering or wound dressing, nanofiber diameter is one of the most important characteristics for the nanofiber mesh produced, as it can affect cell morphology, proliferation and mobility. The natural ECM is a nanofibrous structure that is composed of fibers that have a thickness between 260 nm and 410 nm. Control over the nanofiber diameter offers promising possibilities to be able to better design tissue scaffolds, as cells can distinguish between differently sized nanofibers and will respond accordingly. For example, for keratinocytes, thicker nanofibers (300–700 nm) stimulate cell proliferation, whereas thinner nanofibers promote the cell mobility that is vital for wound closure [43]. For additional stimulation and synergistic effects to nanotopography for dermal wound healing, nanofibers can be incorporated with platelet-rich plasma, which stimulates keratinocyte and dermal fibroblast proliferation [44].

According to the literature, when the aqueous polymer solutions are electrospun, there are two parameters that are the most important for the fine-tuning of nanofiber diameter: total polymer concentration, and relative humidity [45]. In the present study, we were focused on finding the most critical solution parameter, and here the relative humidity remained constant (~17%) for all of these electrospinning experiments. To achieve such low relative humidity, we needed to increase and maintain the temperature at ~37 °C. We have shown here that, in addition to polymer concentration, the PEO proportion and PEO M_w_ impact the nanofiber diameter (Figure 8). Beads, beads-on-a-string, and nanofibers can be obtained by gradually increasing the total polymer concentration (from 2.5% to 4.5%) and the PEO proportion in the dry polymer mix, both of which are related to the increasing viscosity. With further increasing viscosity of the polymer-blend solution, the electrospinning leads to the formation of microfibers with either smooth or rough surfaces. On the other hand, as shown in our models, the polymer-blend solution composition also affects its conductivity, and, through this, the nanofiber diameter. Our data are in line with the literature, where other studies have reported that higher solution conductivity leads to the formation of thinner nanofibers, due to the increased charge density in the ejected jet, which imposes greater elongation forces [20,46].

To sum up, the present study has shown that the nanofiber diameter can be fine-tuned by varying these three polymer-blend solution composition parameters, to obtain nanofibers with diameters from 100 nm to 1000 nm, as well as microfibers, if needed—all of which are composed of >85% alginate.

## 5. Conclusions

This study reports on a thoroughly investigated set of experiments that were designed to produce nanofibers with an alginate content >85% when blended with only high M_w_ PEO as the co-polymer. RSM was used to define the correlations between the polymer-blend solution compositions, its rheological and conductivity parameters, and its electrospinnability and the nanofiber diameters. These nonmodel and RSM analyses showed that the polymer-blend solutions should be conductive, shear-thinning fluids, with greater plastic than elastic behavior, at increasing shear. However, this last one is necessary for appropriate jet formation and stabilization, to survive the stretching, acceleration and whipping of the jet. PEOs of >1 MDa M_w_ improve the electrospinnability of alginate; however, 8 MDa PEO was less appropriate for nanofiber production, and usually resulted in microfibers. The use of 2 MDa and 4 MDa PEOs enables the increase of the mass fraction of the natural polyelectrolyte, which results in nanofibers with a high content of a biodegradable polymer that can provide an efficient scaffold material. The RSM modeling revealed that the nanofiber diameter can be fine-tuned by varying the total polymer concentration of the polymer-blend solution and the PEO proportion in the dry polymer mix. Although the model is applicable only under the experimental conditions defined for the experimental design here, the potential of RSM for the modeling of the blend composition of such a multicomponent solution is promising, and the data provided here serve as a starting point for the design of completely biocompatible nanofibers with >85% polysaccharide content.

## Supplementary Materials

Supplementary Information is available online at https://www.mdpi.com/2073-4360/11/4/692/s1 and contains the remaining figures that are not included in this article, which consists of: Figure S1. Three-dimensional experimental design space with the noted polymer-blend formulations that were successfully electrospun. Figures S2–S6. The remaining cases with the SEM images and the corresponding rheology data. Figures S7 and S8. The surface response analysis of the complex viscosity and loss moduli. Figure S9. The SEM images and the corresponding rheology data of the electrospun PEO solutions.
