# Supplementary material for: Effect of Solution Composition Variables on Electrospun Alginate Nanofibers: Response Surface Analysis
Source: Polymers (Basel). 2019 Apr 16;11(4):692. doi: 10.3390/polym11040692 (PMC6523165; doi:10.3390/polym11040692)
Supplement: Supplementary file 1 [file polymers-11-00692-s001.pdf]

polymers

MDPI

Supporting Information

Effect of Solution Composition Variables on Electrospun Alginate Nanofibers: Response Surface Analysis

Janja Mirtič, Helena Balažic, Špela Zupančič, and Julijana Kristl*

Faculty of Pharmacy, University of Ljubljana, Aškerčeva 7, 1000 Ljubljana, Slovenia

1. Three-Dimensional Representation of Results

Here shown is the same cube from the design of the experiment (Figure 1) with colored formulations with regard to the outcome (non, beads, nanofibers, microfibers). It is seen that there is no direct logic (Figure S1).

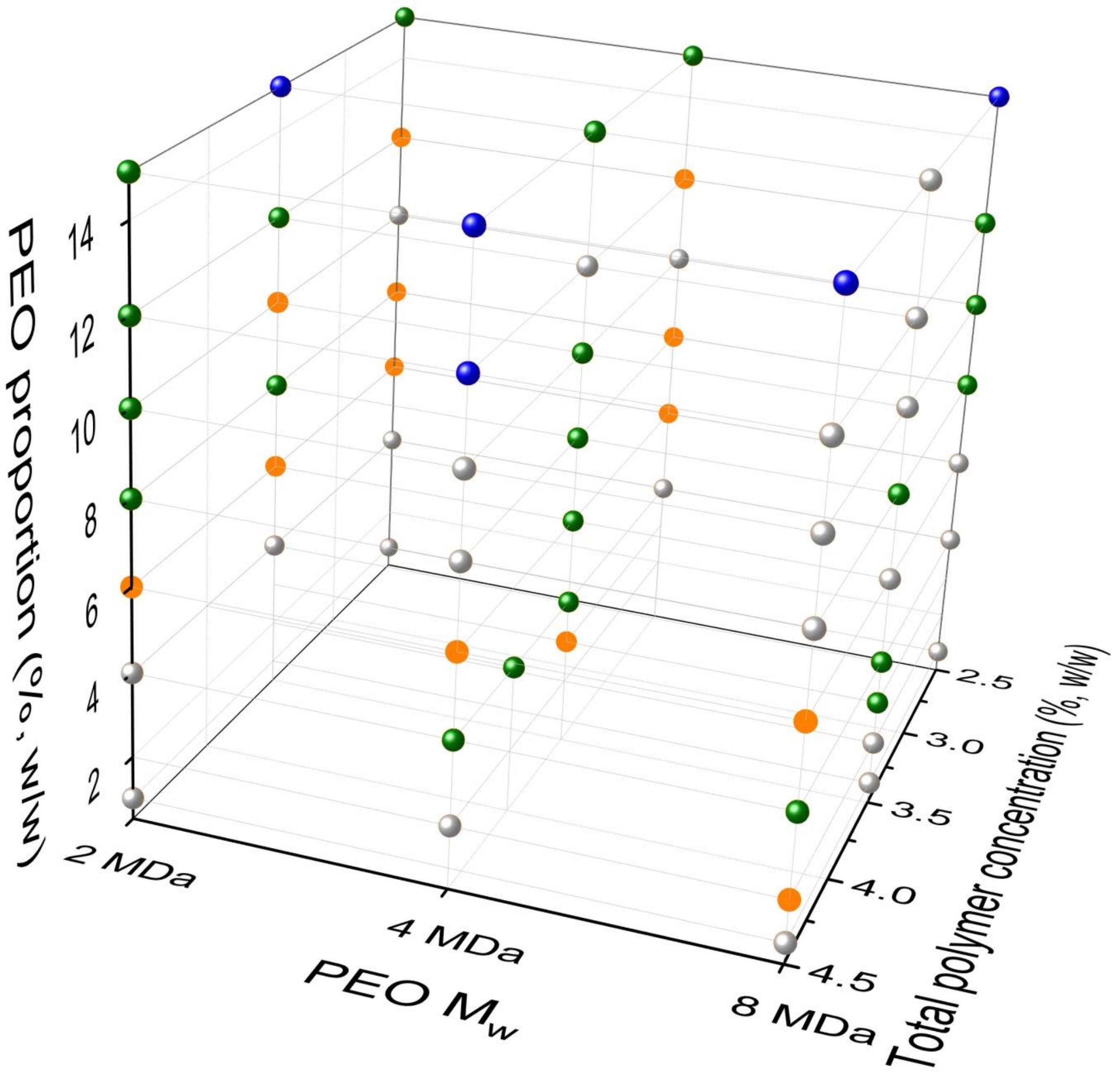

Figure S1. Three-dimensional experimental design space of the polymer-blend solution compositions for the nanofiber formation. The three components varied were: PEO molecular weight (M_w), total polymer concentration and PEO proportion. Legend: green – nanofibers, yellow – beads, blue – microfibers and grey – no sample/could not electrospin.

2. Non-Model Approach to Characterization

2.1. Effects of PEO Molecular Weight

Cases with fixed values of total polymer concentrations and PEO proportion in the dry polymer mix and changing PEO Mw.

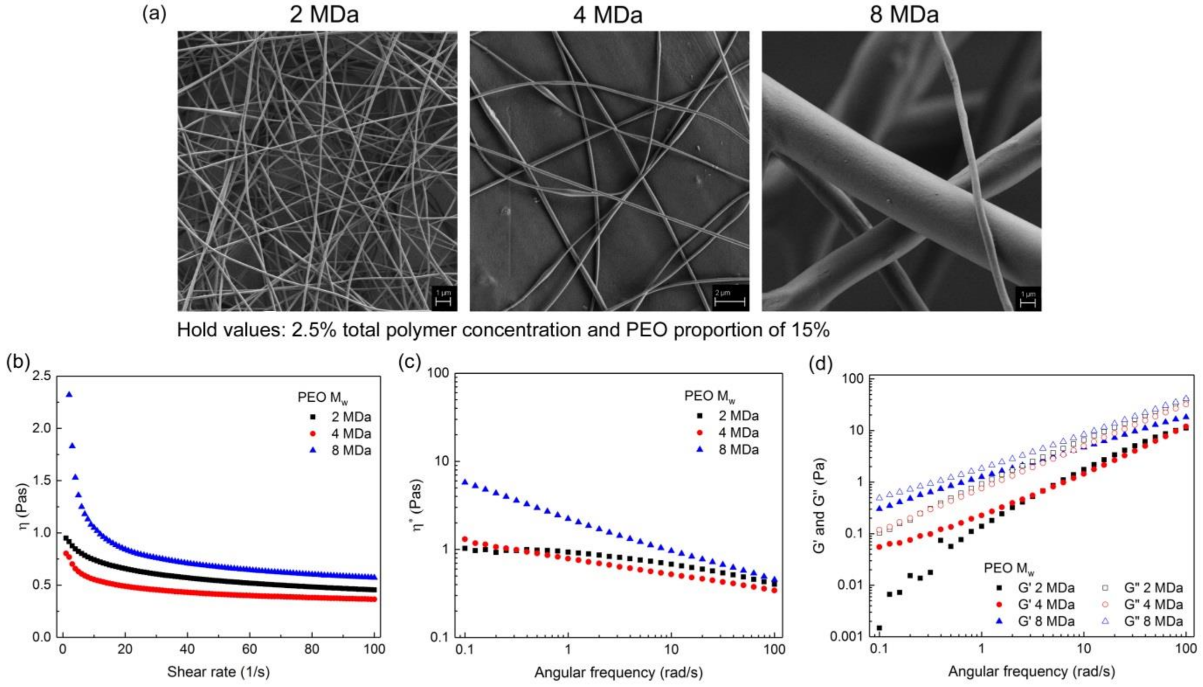

Figure S2. (**a**) Scanning electron microscopy images of nanofibers obtained from the polymer-blend solutions and three different PEO M_w (as indicated), with 2.5% total polymer concentration and PEO proportion of 15%. (**b-d**) Corresponding viscosities (η) (b), complex viscosities (η^*) (c), and storage (G') and loss (G'') moduli (d).

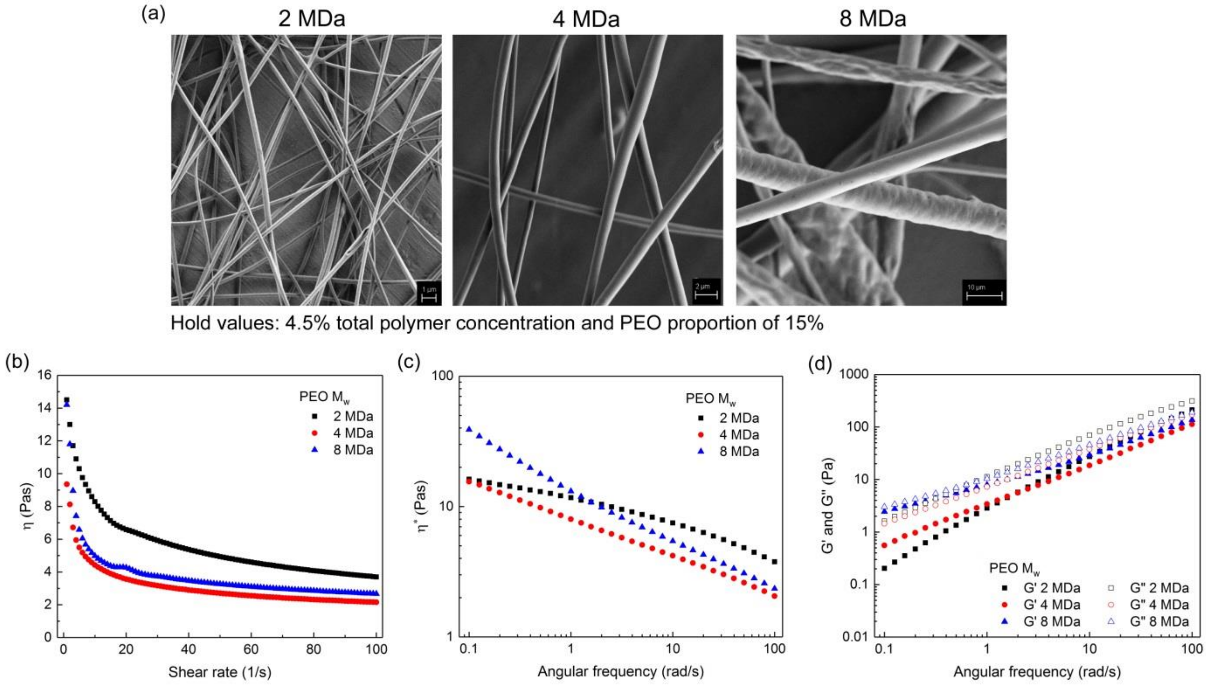

Figure S3. (**a**) Scanning electron microscopy images of nanofibers obtained from the polymer-blend solutions and three different PEO M_w (as indicated), with 4.5% total polymer concentration and PEO proportion of 15%. (**b-d**) Corresponding viscosities (η) (b), complex viscosities (η^*) (c), and storage (G') and loss (G'') moduli (d).

2.2. Effects of Total Polymer Concentration

Remaning case with fixed values of PEO M_w and PEO proportion in the dry polymer mix and changing total polymer concentrations.

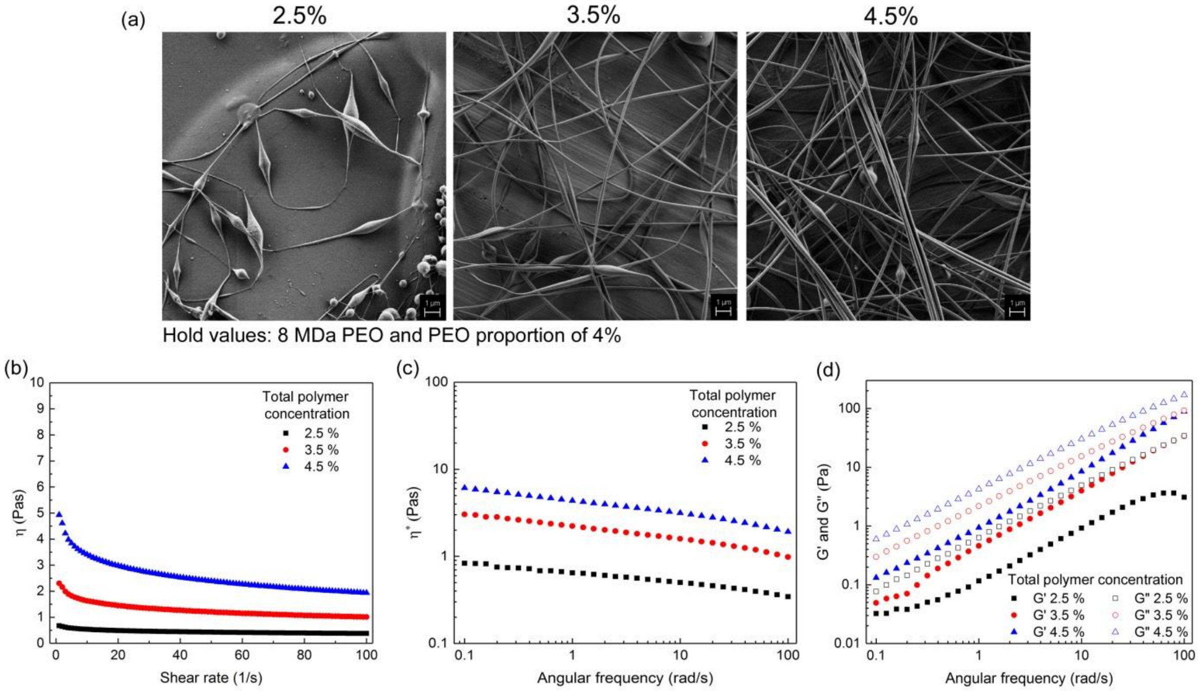

Figure S4. (**a**) Scanning electron microscopy images of nanofibers obtained from polymer-blend solutions with increasing total polymer concentrations (as indicated), with 8 MDa PEO and PEO proportion of 4%. (**b-d**) Corresponding viscosities (η) (b), complex viscosities (η^*) (c), and storage (G') and loss (G'') moduli (d).

2.3. Effects of PEO Proportion in the Dry Polymer Mix

Remaning cases with fixed values of total polymer concentrations and PEO M_w and changing PEO Proportion in the Dry Polymer Mix.

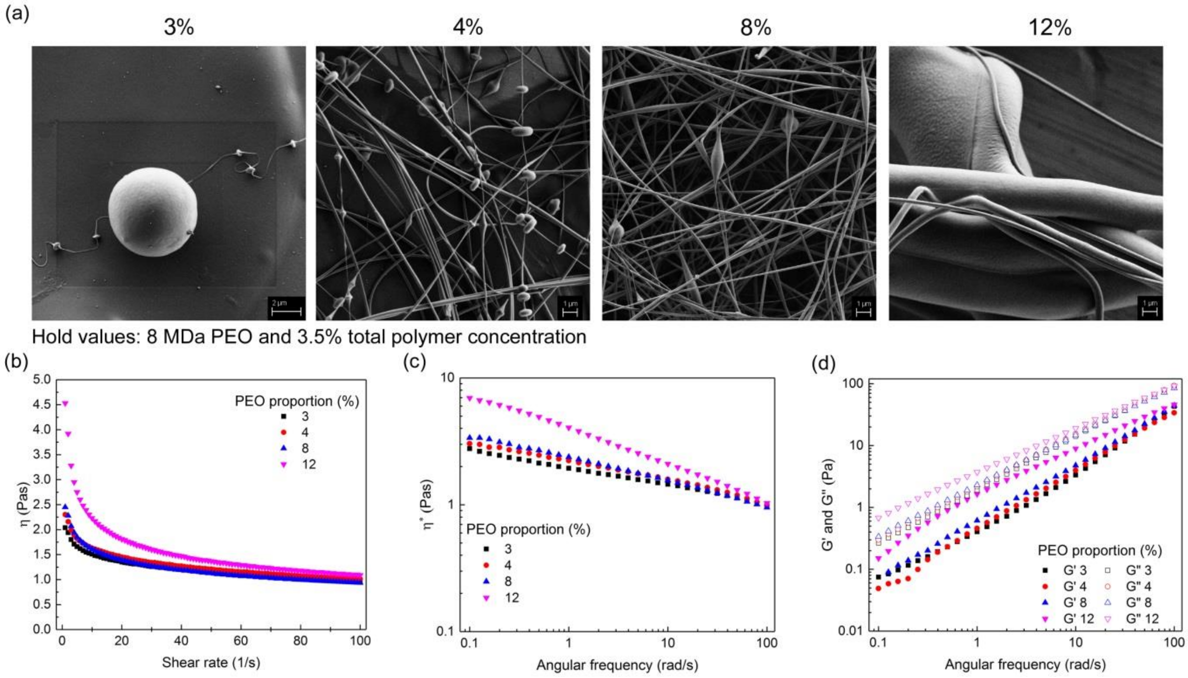

Figure S5. (**a**) Scanning electron microscopy images of nanofibers obtained from polymer-blend solutions formed with different PEO proportions in the dry polymer mix (as indicated), with 8 MDa PEO and 3.5% total polymer concentration. (**b-d**) Corresponding viscosities (η) (b), complex viscosities (η*) (c), and storage (G') and loss (G'') moduli (d).

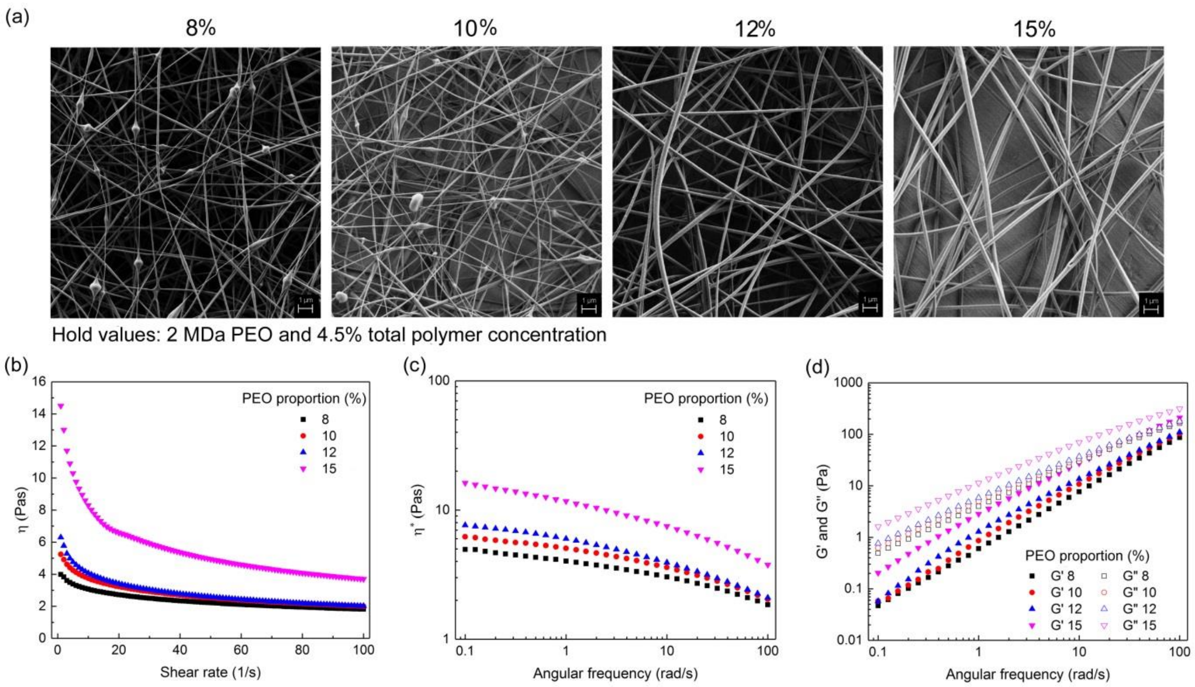

Figure S6. (**a**) Scanning electron microscopy images of nanofibers obtained from polymer-blend solutions formed with different PEO proportions in the dry polymer mix (as indicated), with 2 MDa PEO and 4.5% total polymer concentration. (**b-d**) Corresponding viscosities (η) (b), complex viscosities (η*) (c), and storage (G') and loss (G'') moduli (d).

3. Model Based Approach

Complex viscosity measured in oscillatory mode at 100/s can also we described with the equation of the model (S1) (R^2 = 0.7581, R^2(adjusted)= 0.7178)

$$\eta^* = -0.53 - 0.2 * C2 - 0.0581 * C3 - 0.074 * (C2)^2 + 0.00096 * (C3)^2 + 0.0226 * C2 * C3 \quad \text{(S1)}$$

Complex viscosity is describing similar characteristics as the bulk viscosity and also results are showing the same. With the total polymer concentration (C2) having the biggest effect, with smaller PEO proportion (C3) contribution (Figure S7 a,b,c).

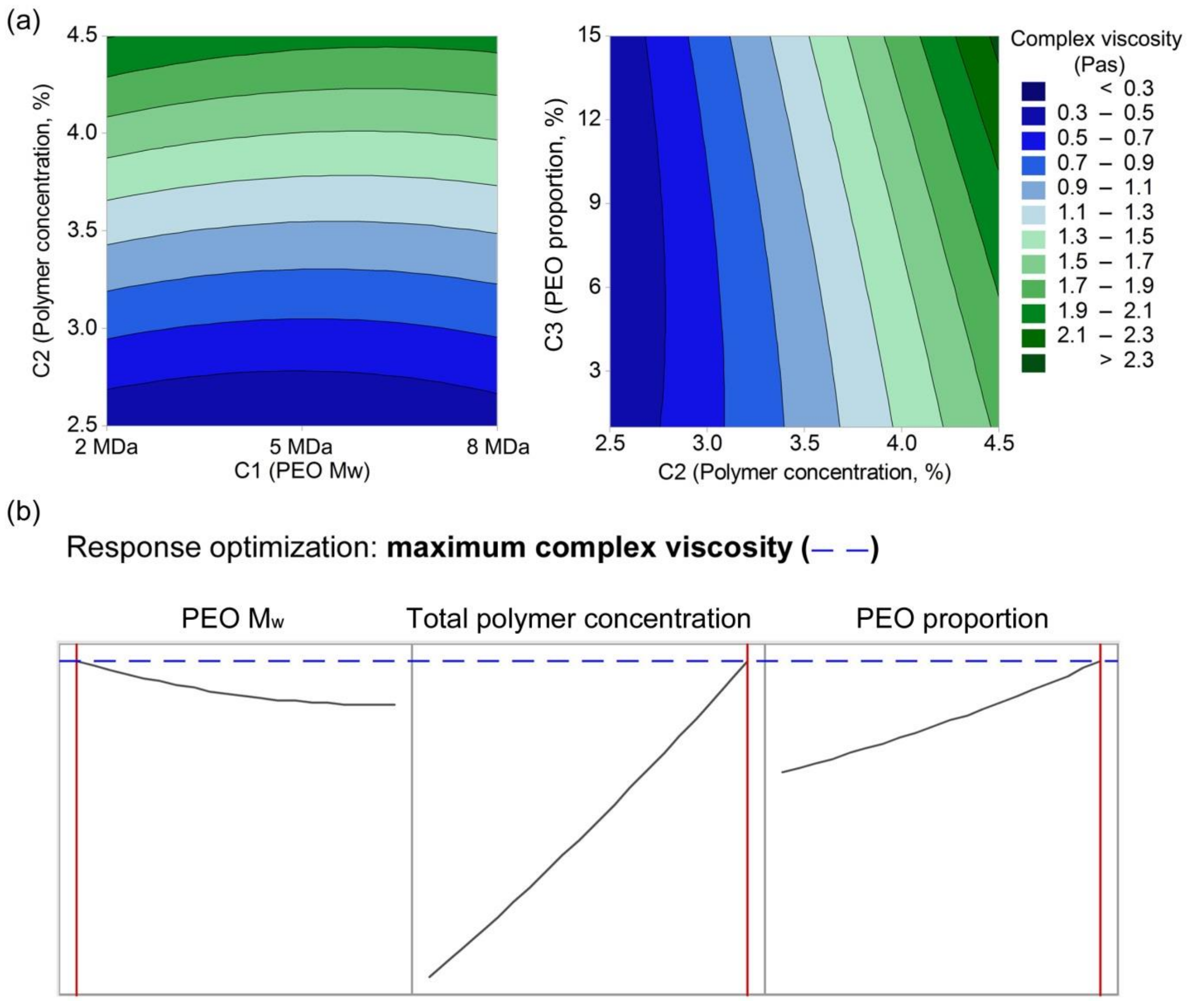

Figure S7. (**a**) Contour plots of the polymer-blend solution complex viscosity at 100 /s as a function of total polymer concentration and PEO M_w (left) and total polymer concentration and PEO proportion (right). (**b**) Response optimization plots of the polymer-blend solution bulk viscosity as a function of PEO M_w, total polymer concentration, and PEO proportion.

Storage modulus can also be described with the equation of the model (S2), however the fit is very poor: $R^2 = 0.3691$, R^2(adjusted) = 0.2659.

$$G' = -9.1 - 0.000004 * C1 + 10.4 * C2 - 2.28 * C3 - 1.41 * (C2)^2 + 0.0299 * (C3)^2 + 0.531 * C2 * C3 \text{ (S2)}$$

Loss modulus can be described with the equation of the model (S3) ($R^2 = 0.8510$, R^2(adjusted) = 0.8266) with a very good fit.

$$G'' = 23 - 0.000002 * C1 - 13.1 * C2 - 2.28 * C3 + 3.41 * (C2)^2 + 0.0695 * (C3)^2 + 0.604 * C2 * C3 \text{ (S3)}$$

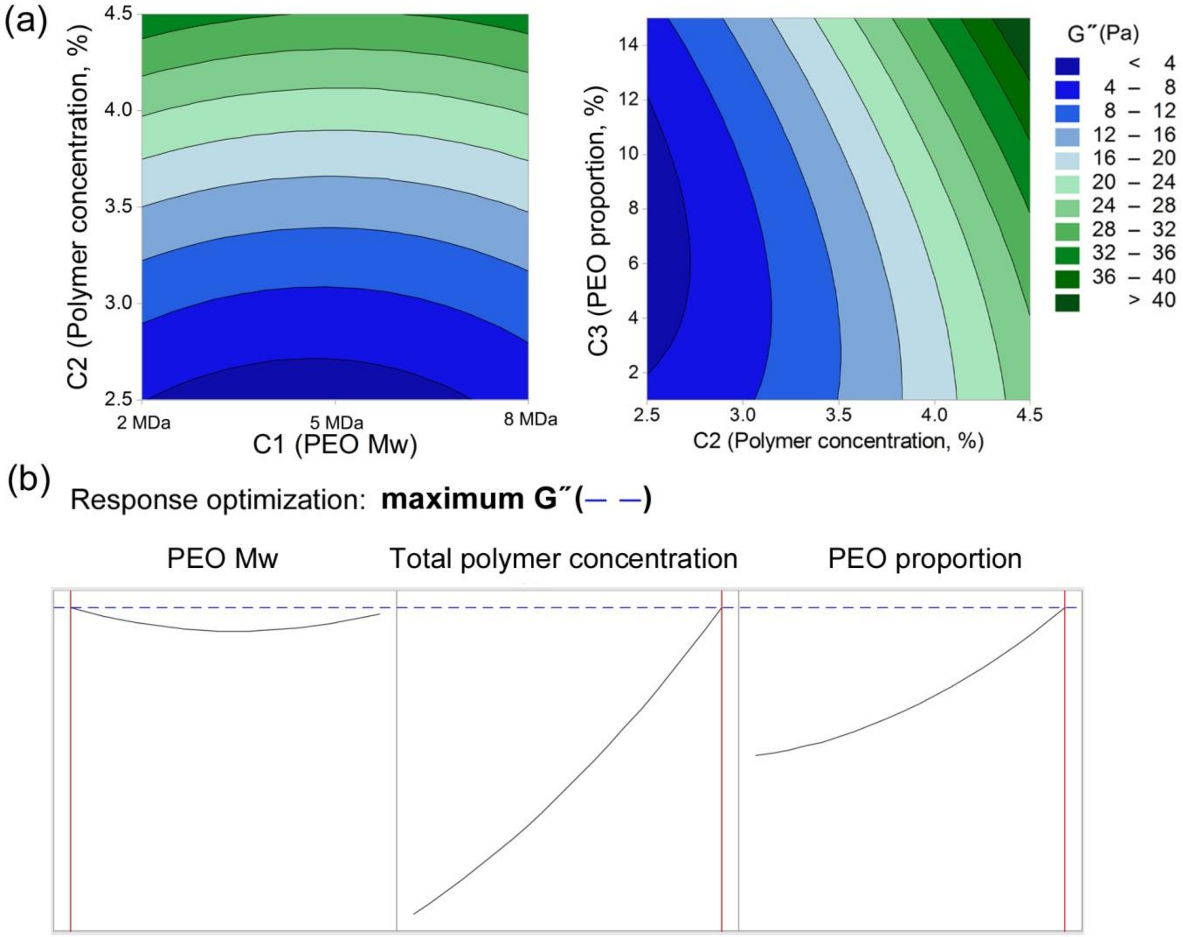

Figure S8. (**a**) Contour plots of the loss modulus as a function of total polymer concentration and PEO M_w (left), and PEO proportion and total polymer concentration (right). (**b**) Response optimization for the loss modulus as a function of PEO M_w, total polymer concentration, and PEO proportion.

4. Electrospinning of PEO Only Polymer Solutions

In order to explain the role of PEO as a carrier co-polymer, we electrospun some of the PEO-only polymer solutions. Here presented are the results of three 2MDa PEO solutions with increasing concentration: 1.05%, 1.5%, and 3.0% (Figure S1). These are all concentrations rather higher than the ones used in alginate blends (where the highest concentration used was 0.675% - calculated from 4.5% x 0.15 (PEO proportion 15%). With lower concentrations of PEO we could not obtain nanofibers, already 1.05% PEO solution results in beaded structures, with 1.5% 179 nm fibers and with 3% 418 nm nanofibers, both having some irregularities on the fiber surface (Figure S1).

These graphs are useful for the comparison as well as an explanation that the PEO is not contributing to the viscosity alone (viscosity curves are rather low).

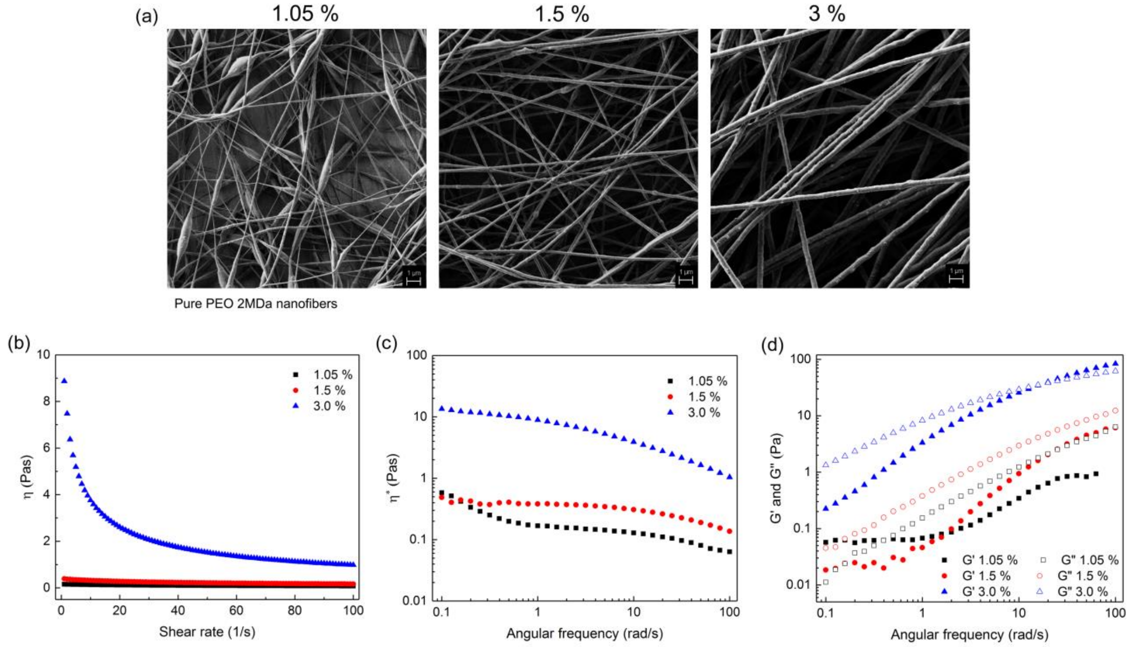

Figure S9. (a) Scanning electron microscopy images of nanofibers obtained from 2MDa PEO solutions in different polymer concentrations; and corresponding (b) viscosity (η); (c) complex viscosity (η*) and (d) loss (G′) and storage (G″) modulus.
